# Retrospect of American Medical Intelligence

**Published:** 1851-02

**Authors:** 


### RETROSPECT

OF

### AMERICAN MEDICAL INTELLIGENCE.

---

*The Speculum Uteri.*—The New York Journal of Medicine, for January, 1851, contains an excellent paper from the pen of Professor Gilman on the use of the speculum uteri, in the investigation of uterine diseases. It is not a little singular that such men as Drs. Lee, Ashwell, Tyler Smith, and Marshall Hall have arrayed themselves against the progress of their profession. Of the invaluable character of the speculum in the diagnosis of female diseases, none who have paid any attention to its practice can entertain any doubt, and we feel pleased with the manly stand that Dr. Gilman has taken on the subject. He sustains himself very admirably, and deserves the thanks of the profession for the clear and convincing views he has given upon the use of the speculum. The following are the closing remarks of Dr. Gilman:

"Dr. Bennett, Dr. Locock, Dr. Murphy, the hundreds, might I not say thousands of practitioners who, in Great Britain, on the continent of Europe, and this country, a part of whose daily business it is to see, and treat, *and cure* these ulcerations, are laboring under some strange hallucination, for to this we are driven—there is no room for mistake. I see a case of chronic leucorrhea; the patient is incapable of exertion, her health is broken, her spirits depressed to the lowest point, for she has been for years under treatment—has tried tonics without number, and washes without end, and all to no effect; I use the speculum. I *think* I see the cervix large, red, and on either lip I *imagine* that I see an ulcer. Under the influence of this idea—supposing, nay, so far has the delusion gone with me, verily believing that there is an ulcer—I apply nitrate of silver; I cauterize this *supposed* ulcer. I do this again and again; my patient gradually improves in health—is convalescent—is well. She returns to her home, a happy, useful wife; and in a year or two I get a letter, full of gratitude and happiness—the long barren wife is a mother. Now is this case—and, like many others who devote special attention to diseases of the uterus, *and use the speculum,* my experience will supply many such—is this case a mere delusion? Have I dreamed that I saw this ulcer—that, after repeated cauterizations, I saw it growing less and less, till nothing but sound mucous membrane appeared? Have I dreamed all this? Must I, at the dictum of Dr. Lee, or Dr. Ashwell, or Dr. any-body-else, give up my own observation, my own experience? And why? Because they will not look, and do not see. But there are great moral considerations, and Dr. Ashwell says he should feel tempted to give up the treatment of the diseases of women altogether, if the speculum continued to be used as it has been. And shall I follow his example? shall I give up the instrument that has enabled me to restore scores of women to health and happiness, because it is indelicate? Well may Dr. Locock say that the talk about the indelicacy of the use of the speculum was all nonsense. Nonsense it is—poor, paltry, but yet mischievous, most mischievous nonsense; and, for myself, I believe that all this talk about treating the use of the speculum is little better, and most of that about its abuse is very much worse. The attempt to cast reproaches on honorable men for well-intended efforts to advance our science and improve our means of curing disease, because those attempts involve personal exposure, is much more likely to have its origin in professional jealousy than in moral principle."

*Tetanus with recovery.*—Dr. Lente, resident Surgeon of the New York Hospital, reports in the New York Journal of Medicine, a very interesting case of tetanus, after a compound fracture of the metacarpus. The tetanus continued for fifty-three days, and was managed with laudanum, antimonial tartar, ether, by inhalation, enemata of assafetida, tobacco cataplasms, croton oil, morphine, etc. The patient recovered, and was discharged, on the fifty-third day, cured.

*Hydrocele.*—Dr. Lente also reports a case of hydrocele, from the tumor of which forty-two ounces of water were drawn off in the first operation. Dr. Post has removed three quarts of fluid at one time.

Notwithstanding the size of the tumor, in Dr. Lente's case, he resorted to the injection of tinct. iodine one part, water three parts, with beneficial results.

---

*Ascites.*—Dr. T. D. Lee, of New York, reports, in the New York Journal of Medicine, a case of ascites, in which the operation of Paracentesis Abdominis was performed thirty-nine times, and one hundred and forty-one gallons of water were removed. The patient died on the 3d of July, 1850.

---

*Dr. Davis's History of Medicine.*—Our confrère of the New York Journal of Medicine gives Dr. Davis a severe castigation for the work dignified with the title of History of Medicine. We think the profession geneally will coincide with the sentiments of the reviewer; Dr. Davis's History of Medicine is neither a work of information nor of accuracy. Instead of being a history of American Medical Institutions, it is a gross caricature of the subject. It proclaims deficiency where none exists, and assails the profession in a style that is neither truthful nor useful. The man who rises from the perusal of Dr. Davis's History, under the impression that he has read the details of American medical history, will make as great a mistake as he would in supposing he had read the history of America, in reading Paulding's History of John Bull and Brother Jonathan.

The united efforts of medical men should be directed to the elevation of professional excellence, an object not likely to be promoted by such works as the one which Dr. Davis courteously calls a "History of Medical Education and Institutions in the United States."

---

*Chloroform an Antiseptic.*—M. Augend, of Constantinople, has transmitted to the Academy of Sciences, Paris, the following experiment with chloroform:

"Take three wide-mouthed flasks, the first containing a few drops of ether, the second a few drops of chloroform, the third left empty. If in each of these a piece of beef be placed, and the flasks be closed and left undisturbed in the summer season, the following circumstances will be observed:—The meat, which was of a reddish brown color in its natural state, changed instantly to a vermilion-red in the mixture of chloroform and air, while in the ether vapor no change occurred. At the end of a week the difference was greater still; the meat in the flask containing atmospheric air was but little changed in its aspect; that in chloroform had acquired the appearance of boiled meat. On opening the flasks it was found that the meat, both in the atmospheric air and in the ether vapor, was putrefied, and emitted a most offensive odor; while that in the mixture of chloroform and air had the sweetish taste and odor of chloroform.

"M. Augend has ascertained that 1.200th of chloroform completely prevents the putrefaction of fresh meat.

The most apparent action of the chloroform is the rapidity with which it traverses the thickest tissues, and causes an immediate contraction of their parenchyma, with consequent exudation of the fluids of the structure experimented upon. The author further dwelt upon the value, in a medico-legal point of view, that chloroform thus possesses in arresting putrefaction."

Admitting the full truth of these statements, we see no special gain to the profession in the facts. We have in arsenic and corrosive sublimate all the requisites for antiseptic purposes that are needed, for no one will pretend that chloroform is superior to these agents, and they are much cheaper.

---

*On the Use of Therapeutic Agents in relieving Rigidity of the Os and Cervix Uteri.*—Dr. Archibald Hall, in the British American Medical and Physical Journal, makes the following remarks:

"Therapeutic agents are of value exactly in accordance with the effects which, under peculiar circumstances, they are capable producing. This I apprehend to be an axiom in medicine, and by which the relative value of medicinal agents may be accurately measured. I propose now to examine these several modes of treatment, keeping the above axiom in view.

"1. With regard to venesection. This seems to be the established rule of practice, and is sanctioned by every author of note. Bleeding, in its influence upon the system, is one of the most certain and powerful sedatives which we possess, and presents strong claims to our consideration, under the peculiar circumstances of the case. To be of any real value, however, it requires to be vigorously practiced, and relaxation will be found rarely to follow, until a large quantity of blood has been abstracted, and syncope is on the point of supervention. There can be no question that the best effects follow its employment, as far at least as rigidity is concerned. But is this line of practice always expedient? Is it always imperiously demanded? When the os uteri exhibits heat, dryness, and tenderness, such effects being the evidence of existing inflammation, the propriety of the practice cannot be questioned. But before the development of these symptoms, several hours may elapse, during which the effects of irritation alone are apparent as indicated by the mere rigidity. Under these circumstances, bleeding *is not* imperiously demanded, chiefly because it entails an unnecessary withdrawal of blood, inductive of debility, and protracted recovery—thus effecting more than we desire. The practice, however, is recommended and sanctioned by Burns, Dewees, Blundell, Ramsbotham, Chailly, Cazeaux, and a host of others.

"2. The exhibition of opium is not always attended with the advantages which we might, *a priori*, be led to expect from its well known narcotic powers. If exhibited, full doses should be employed; but, as in the case of bleeding, it is liable to effect too much—it may lull the uterine contractions, and thus suspend the labor, which it is generally desirable to expedite.

"3. The local application of belladonna. This was first proposed by Chaussier, who suggested its use in the form of ointment made with cerate, reasoning analogically from the influence of this medicine upon the iris. Dubois subsequently used the extract in its undiluted state. The practice is peculiarly French, and has not been followed to any extent by either British or American accoucheurs. That the application of the belladonna will produce relaxation is admitted on all hands; but the extent of that relaxation cannot be predetermined. It may affect the whole muscular coat of the uterus, and be thus productive of alarming consequences.

"4. Tartar emetic. The prostration and muscular relaxation produced by this agent, almost naturally indicate its employment in cases of the kind we are considering. Nausea having been once established, the rigidity will in a very large majority of cases be found readily to yield. Tartar emetic seems almost to exert a special influence on the cervix, for while the contractions of the fundus and body of the uterus are not interfered with, dilatation of the cervix will be found to proceed rapidly, and this the result of the reëstablishment of the reflex actions existing between the stomach and the uterus, which are apparently suspended.

"The few authors who have advocated the employment of tartar emetic in these cases, have generally prescribed it in doses of one-fourth of a grain repeated every three or four hours, until its influence became apparent by the gradual dilatation of the mouth of the uterus. There is certainly no worse system of midwifery than the meddlesome midwifery; but if, in any case, interference is demanded, whether of a manual or medicinal nature, the object should be a speedy delivery, consistent with the safety of the mother and the child. My own observation has led me to the belief that it may be safely resorted to in much larger doses, and with more prompt relief; and I cannot but view the mode of employing the medicine indicated above, as attended with a very considerable and quite unnecessary sacrifice of time on the part of the accoucheur, and of prolonged suffering on the part of the patient. I, accordingly, prescribed it in one grain doses, repeated every half hour, and I have not as yet found it necessary to exhibit the medicine in such doses more than thrice, relaxation most commonly following the exhibition of the first grain."

---

*Professor Paul F. Eve.*—This gentleman terminated his lecture term in the Louisville Medical School on Friday, the 31st January, and started for Georgia on the 1st day of February. The connexion of Professor Eve with the Louisville School has been an eminently satisfactory one, and before his departure for the South, Professor Eve received the most gratifying testimonials from all of his colleagues, the Board of Trustees of the University, and from the medical class, of the high appreciation placed upon his services. Nor was this appreciation confined to the sources named; the practicing portion of the profession in the city, entertain for Prof. Eve those sentiments of respect and esteem which are commanded only by true merit. He has won a high position in Kentucky, and we earnestly hope he may long continue to retain it. B.

---

*Prof. Silliman.*—The friends of the Louisville Medical School will be pleased to learn that Professor Silliman is about to visit Europe, where he will remain until next Autumn. The Trustees of the University, and the colleagues of Professor Silliman have placed at his disposal means for enlarging the teaching facilities of the school, and from what we know of Professor Silliman's intentions we can safely promise a considerable addition to the already ample resources of the school. There are few persons to whom such a trust as this could be confided with an equal measure of confidence in the judiciousness of the expenditure. B,

---

*Auvert's Plates.*—Among the recent acquisitions to the surgical department of the Louisville Medical School, we have examined the great work of Auvert, Imperial Surgeon of Russia, with more than ordinary satisfaction. The plates are superior to anything we have ever seen in surgical works, and in order to secure their perfection and keep the selling price within reasonable limits, the Emperor Nicholas contributed 60,000 silver rubles towards the expense of the work. It seems to us that these plates have reached the highest perfection of art; they seem to be the very presentment of the diseased conditions they are intended to pourtray.

One half of this able work has reached this city, and we have enjoyed an unusual degree of pleasure in examining its merits. A careful study of the plates cannot fail to be of great service to students of medicine in preparing themselves for the practice of their profession, and no one who may fix the features of these plates in his memory can ever regret the labor and time bestowed on their study.

The following is a list of the subjects delineated in these masterly engravings:

Tabula 1.—Os Frontis Meliceride perforatum.
" 2 and 3.—Calvariæ Fractura.
" 3.—Carie Syphilitica Calvariæ basis exesa.
" 4.—Dermatokeras frontis.
" 5.—Polypus sinus frontalis dextri.
" 6.—Elephantiasis dura dextræ — angiectasi integumentorum cranii vicinorum juncta.
" 7.—Elephantiasis integumentorum occipitis.
" 8 and 9.—Fungus hæmatodes oculi dextri.
" 9 and 10.—Fungus Encephaloideus oculi dextri.
" 10 and 11.—Fungus Melanodes oculi sinistri.
" 11—contains three beautiful figures, which are in the very highest style of art.
" 12 and 13.—Canceri palpebræ inferioris sinistræ.
" 14.—Angiectasis apicis nasi—beautifully executed—we speak surgically.
" 15.—Angiectasis genæ sinistræ.
" 16.—Lipoma genæ dextræ concrementis calcariis farctum.
" 17 and 18.—Osteo-sarcoma ingens maxillæ superioris sinistræ and dextræ.
" 19, 20, 21, and 22 represent the preceding cases of disease after operations for the removal of the osteo-sarcoma, and speak favorably of the surgical skill of M. Auvert.
" 23.—Osteo-sarcoma symphisi maxillæ superioris insidens.
" 24—Is an admirable Plate, showing the result of an operation in the preceding case.
" 25 and 26.—The first is a formidable case of osteo-sarcoma of the superior maxillary bone. Second—the same subject after an operation.
" 27.—Ingens tumor cysticus regionis parotidæ sinistræ.
" 28.—Steatoma regionis parotidæ dextræ.

" 29.—Lipoma labii superioris ejusque integumentorum angiectasis.
" 30.—Atresiaoris imperfecta,—a beautiful specimen.
" 31.—Cancer nasii.
" 32, 33, 34, 35, 36.—Specimens of osteo-sarcoma.
" 36, 38, 39.—Osteo-sarcoma ingens maxillæ inferioris.
" 40, 41, 42.—Cancer labii inferioris.
" 43.—Cancer condylomatosus anguli oris dextrii.
" 44.—The same subject after an operation.
" 45.—Cancer labii inferioris ex utroque angulo oris proficiscens.
" 46.—A delineation of the above case after an operation.
" 47.—Cancer labii inferioris utramque ejus commissuram afficiens.
" 48.—A Plate of the preceding case after an operation.
" 49.—Cancer ingens labii cum excidio subjacentis maxillæ inferioris.
" 50.—Steatoma regionis parotidæ dextræ.
" 51.—Ingens lipoma colli.
" 52.—Ingens hygroma cervicis.
" 53.—Aneurisma trunci carotidis sinistræ.
" 54.—Delineates very beautifully and clearly the various stages in the performance of the operation for the preceding case.
" 55.—Aneurisma arteriæ subclavicæ dextræ supra claviculam situm.
" 56.—Surgical anatomy of the preceding regions.
" 57.—Large aneurism, right subclavian, infra claviculam situm.
" 58.—Anatomical description of the preceding operation.
" 59.—Aneurisma subclavian dextræ infra claviculam.
" 60.—Anatomical description of the operation for the preceding case.

We wish it were possible to convey a correct knowledge of the excellence of these Plates, for we feel very certain that wherever their merits are known, there will be a desire to examine and study them. B.

---

*Jarvis's Adjuster.*—In the October No. of this Journal, for 1850, we called the attention of medical readers to the merits of Jarvis's Adjuster, in the reduction of dislocations. The views we then gave of the instrument have been amply confirmed since that time, and, if possible have gained new strength. We have used the Adjuster in two cases of dislocation of the shoulder, and its vast superiority over the pullies, and over all other means of reduction we have seen, was very manifest. In the first of the two cases, we were requested to visit a fracture of the arm, in the night, and we went without the instrument. The case was found to be a severe dislocation of the shoulder, caused by a fall through an open cellar door, the shoulder striking a barrel standing on its end, on the floor of the cellar. There was a great degree of violence done to the soft parts, and the head of the humerus was driven into the axilla. The subject of the injury being some distance from the instrument, reduction was attempted by the aid of a number of men, and for a while eleven men were engaged in extension and counter-extension. But the bone was immoveable. The Adjuster was sent for and applied, and in a short space of time, and with but little suffering on the part of the patient, the reduction was effected. We are confident that the pullies would have required a greater length of time, and would have inflicted more suffering than the Adjuster.

A few days since a large, muscular boatman called at the office of the writer, for the purpose of having a serious dislocation of the shoulder reduced. The bone was driven deep into the axilla, and it had resisted the attempts that had been made at reduction. The subject of this dislocation had suffered with the evils of a former one, and he dreaded the attempt at extension and counter-extension. The Adjuster was applied, and a few circles of the pinion wheel restored the bone to its place with an audible snap, and the patient was so much delighted that he declared that a man with a dislocation of the shoulder might afford to walk fifty miles to secure the services of such an instrument as the Adjuster, rather than submit to the ordinary means of reduction.

Nothing could induce us to be without this instrument; with its aid dislocations are disarmed of the annoying character they once possessed. B.

---

*American Medical Association.*—We copy the following from the Charleston Medical Journal:

"The Committee of Arrangements request all societies and other institutions authorized to send delegates, to forward a correct list of those selected to attend the next annual meeting, to the Secretary, Dr. H. W. DeSaussure, at Charleston, S. C., on or before the first day of April.

"In consequence of the resignation of Dr. Stillé, one of the Secretaries, from ill health, all communications intended for the next meeting of the Association must be addressed to the remaining Secretary, Dr. H. W. DeSaussure, Charleston, S. C.

"The Fourth Annual Meeting of the American Medical Association will be held at Charleston, S. C., on the 1st Tuesday of May next.

"Editors of Medical Journals will please give the above notice an early insertion in their respective journals."

There was an error in the above announcement of the Charleston Medical Journal, which we have corrected, the more especially as the error has passed unchanged into a number of the Medical Journals. The error is in stating that the meeting of the Medical Association is to be held in Charleston, S. C., on the *second* Tuesday, (13th) of May. It should be the *first* Tuesday, (6th) of May. The meetings of the Association are regularly held on the first Tuesday in May, and the fact should be extensively known, or some of the delegates may get to Charleston after the adjournment. B.

